# A phase I trial of antibody directed enzyme prodrug therapy (ADEPT) in patients with advanced colorectal carcinoma or other CEA producing tumours

**DOI:** 10.1038/sj.bjc.6600517

**Published:** 2002-09-09

**Authors:** R J Francis, S K Sharma, C Springer, A J Green, L D Hope-Stone, L Sena, J Martin, K L Adamson, A Robbins, L Gumbrell, D O'Malley, E Tsiompanou, H Shahbakhti, S Webley, D Hochhauser, A J Hilson, D Blakey, R H J Begent

**Affiliations:** Cancer Research UK Targeting and Imaging Group, Department Oncology, Royal Free and University College Medical School, University College London, London NW3 2PF, UK; Cancer Research UK Centre for Cancer Therapeutics, Institute of Cancer Research, Sutton, Surrey SM2 5NG, UK; Drug Development Office, Cancer Research UK, PO Box 123, London WC2A 3PX, UK; AstraZeneca, Mereside, Alderley Park, Macclesfield, Cheshire SK10 4TG, UK

**Keywords:** monoclonal antibody, ADEPT, carcinoembryonic antigen (CEA), colorectal neoplasm, clinical trial

## Abstract

Antibody-directed enzyme prodrug therapy is a targeted therapy in which a prodrug is activated selectively at the tumour site by an enzyme, which has been targeted to the tumour by an antibody (antibody-enzyme conjugate). Previous clinical trials have shown evidence of tumour response, however, the activated drug had a long half-life, which resulted in dose-limiting myelosuppression. Also, the targeting system, although giving high tumour to blood ratios of antibody-enzyme conjugate (10 000 : 1) required administration of a clearing antibody in addition to the antibody-enzyme conjugate. The purpose of this current study therefore was to attempt tumour targeting of the antibody-enzyme conjugate without the clearing antibody, and to investigate a new prodrug (bis-iodo phenol mustard, ZD2767P) whose activated form is highly potent and has a short half-life. Twenty-seven patients were treated with antibody-directed enzyme prodrug therapy using A5CP antibody-enzyme conjugate and ZD2767P prodrug, in a dose-escalating phase I trial. The maximum tolerated dose of ZD2767P was reached at 15.5 mg m^−2^×three administrations with a serum carboxypeptidase G2 level of 0.05 U ml^−1^. Myelosuppression limited dose escalation. Other toxicities were mild. Patients' quality of life was not adversely affected during the trial as assessed by the measures used. There were no clinical or radiological responses seen in the study, but three patients had stable disease at day 56. Human anti-mouse antibody and human anti-carboxypeptidase G2 antibody were produced in response to the antibody enzyme conjugate (A5CP). The antibody-enzyme conjugate localisation data (carboxypeptidase G2 enzyme levels by HPLC on tumour and normal tissue samples, and gamma camera analysis of I-131 radiolabelled conjugate) are consistent with inadequate tumour localisation (median tumour: normal tissue ratios of antibody-enzyme conjugate of less than 1). A clearance system is therefore desirable with this antibody-enzyme conjugate or a more efficient targeting system is required. ZD2767P was shown to clear rapidly from the circulation and activated drug was not measurable in the blood. ZD2767P has potential for use in future antibody-directed enzyme prodrug therapy systems.

*British Journal of Cancer* (2002) **21**, 600–607. doi:10.1038/sj.bjc.6600517 www.bjcancer.com

© 2002 Cancer Research UK

## 

Colorectal cancer is the second most common cause of cancer death in the UK. Conventional chemotherapy is unable to cure patients with advanced or metastatic disease, so there is an urgent need to develop novel therapies. Systemic treatments for most solid tumours are limited by lack of specificity and the emergence of drug resistance. Antibody directed enzyme prodrug therapy (ADEPT) is designed to overcome these obstacles to successful therapy (Bagshawe *et al*, 1988).

ADEPT is a two step targeted therapy. The first step involves the administration of an antibody-enzyme conjugate, which targets the tumour. This is followed by the administration of a prodrug, which is activated by the enzyme to form a chemotherapeutic agent at the site of the tumour. Each enzyme molecule can activate many molecules of prodrug, which results in large amounts of drug generated at the tumour (amplification effect). As the activation of prodrug occurs extracellularly, active drug can also diffuse to neighbouring cells, killing them with a bystander effect.

Previous clinical trials of ADEPT have used A5CP, which consists of a F(ab)_2_ fragment of a mouse monoclonal antibody to CEA (A5B7) linked to the bacterial enzyme carboxypeptidase (CPG2), as the antibody-enzyme targeting agent. This was used in combination with a benzoic acid mustard prodrug, CMDA, which is activated by cleavage of its glutamate moiety (Bagshawe *et al*, 1995; Napier *et al*, 2000). These trials have shown evidence of tumour response (Bagshawe *et al*, 1995; Napier *et al*, 2000), however, toxicity was thought to have resulted from the long half-life of the activated drug, which diffused back into the circulation to cause myelosuppression (Springer *et al*, 1993; Martin *et al*, 1997). Also, the targeting system, although giving high tumour to blood ratios of antibody-enzyme conjugate (10 000 : 1), was complicated, requiring additional administration of a mouse monoclonal clearing antibody directed to the active site of CPG2 (Napier *et al*, 2000). This antibody was galactosylated to accelerate its clearance from the circulation (Sharma *et al*, 1990, 1994).

The purpose of this current study was to investigate tumour targeting of the antibody-enzyme conjugate (A5CP) without the clearing antibody, and to study a new prodrug (bis-iodo phenol mustard, ZD2767P) whose activated form has a short half-life and is highly potent. ZD2767P was designed to overcome the problem of long-acting active drug diffusing from tumour into the circulation and causing myelosuppression (Springer *et al*, 1995; Blakey *et al*, 1996). The study was designed to incorporate conventional phase I clinical trial measures, including toxicity and pharmacokinetics, with mechanistic studies in order to achieve a better understanding of ADEPT, and to identify potential improvements for future ADEPT systems.

## MATERIALS AND METHODS

### Materials

A5CP was manufactured by Lonza Biologics, and supplied by AstraZeneca Pharmaceuticals. A5CP was formulated as 50, 159 or 150 unit per ml sterile aqueous solution packed in neutral (Type 1) glass vials with a nominal volume of 5 ml. A5CP was diluted in 0.9% sodium chloride and administered over 2 h as a 500 ml intravenous (i.v.) infusion.

Radiolabelling of A5CP was performed using 370 MBq of ^131^I by the N-bromo-succinamide/L-tyrosine technique (Adam, 1989). Thin layer chromatography (TLC) was performed to assess iodine incorporation and antigen binding was assessed using a CEA column.

ZD2767P was manufactured and supplied by AstraZeneca as a non-sterile product containing 610 mg of crystalline ZD2767P hydroiodide per vial (which was equivalent to 500 mg of ZD2767P free base). Ten ml of sodium bicarbonate solution was added to this and ZD2767P hydroiodide salt was converted to ZD2767P di-sodium salt. The final solution contained 53.7 mg ml^−1^ of ZD2767P di-sodium salt, which was equivalent to 50 mg ml^−1^ of free base. ZD2767P was administered as three bolus doses an hour apart, into a fast running i.v. infusion of Dextrose 5%, through a central (Hickman) line.

## Methods

### 

#### A5CP Pharmacokinetics

Blood samples were taken at 5 min, 1, 2, 3, 5 h and then daily after A5CP administration to assess clearance of A5CP from blood. The catalytic activity of CPG2 in serum was used to prospectively measure clearance of A5CP. This was performed initially by spectrophotometric methotrexate reduction assay (Mcculloch *et al*, 1971; Hughes *et al*, 1982), and later by the semi-automated Cobas Fara II centrifugal analyser (Cobas assay). CPG2 enzyme levels below 0.05 U ml^−1^ were confirmed by a methotrexate reduction assay on HPLC (Blakey *et al*, 1996). The clearance of ^131^I-radiolabelled A5CP was assessed retrospectively by gamma counting of plasma samples. The clearance of A5CP by both CPG2 enzyme levels and ^131^I-radioactivity was then graphed and a biexponential model fitted to calculate alpha and beta half-lives.

#### Serum CPG2 enzyme levels on prodrug day

A blood sample was taken immediately prior to prodrug administration and was analysed retrospectively by methotrexate reduction assay on HPLC to confirm the CPG2 enzyme level in serum.

#### CPG2 enzyme levels in tumour and normal tissue

In patients who gave their consent, tumour or bone marrow biopsies were taken on prodrug day to assess A5CP localisation. These samples were analysed for CPG2 enzyme levels by HPLC using a methotrexate reduction assay (Blakey *et al*, 1996).

#### Gamma camera imaging

Patients who received ^131^I-radiolabelled A5CP were imaged daily on an ADAC Vertex Plus dual headed gamma camera. The imaging consisted of planar and SPECT acquisitions. SPECT images were reconstructed using ADAC filtered-back projection software and were corrected for decay, attenuation and Compton scatter. Analysis was then performed using region of interests placed in areas of tumour and normal tissue (heart, lung, liver) as previously described (Green *et al*, 1990). The per cent injected radioactivity dose per kg was calculated for each of these tissues. The enzyme level (U g^−1^) was estimated in the tumour for prodrug day, by modelling the data as a mono-exponential clearance and extrapolating to prodrug day.

#### Immunogenicity

Blood was taken from patients prestudy and at weekly intervals post A5CP administration for 8 weeks. Human anti-mouse antibody (HAMA) and human anti-CPG2 antibody (HACPG2A) response was measured by ELISA and compared with known positive and negative controls (Sharma *et al*, 1992).

#### ZD2767P (prodrug) pharmacokinetics

ZD2767P prodrug concentrations in plasma were measured by HPLC. Plasma samples were taken for prodrug level estimations 2 min after the first and second prodrug injection, and at 2, 5, 10, 15, 30 and 60 min after the third prodrug injection. A complete pharmacokinetic profile including concentration of ZD2767P extrapolated back to time 0 min (C_0_), volume of distribution in the body at steady state (V_ss_), area under the curve (AUC) and elimination half-life of prodrug (T_1/2_) was determined for each evaluable patient using a non-compartmental model and *WinNonlin* software.

#### Comet assay

The short half-life of the active drug of ZD2767P prevented it being directly measured in the clinical trial. However, as it is an alkylating agent, its lethality to cells is via the formation of DNA interstrand cross-links. The presence of DNA interstrand crosslinks was measured in the trial by a single cell comet assay. This was performed on tumour biopsy specimens and bone marrow aspirates. Peripheral blood lymphocytes taken at the same time as the biopsy were used as controls. All tumour or bone marrow biopsies were performed on the day of receiving prodrug, 1–2 h after receiving the last prodrug injection (Webley *et al*, 2001).

#### Toxicity assessment

Toxicity was assessed using National Cancer Institute Common Toxicity Criteria (NCI-CTC) (National Cancer Institute, 1988).

#### Response assessment

Response was assessed using standard WHO response criteria, based on change in maximal bidimensional diameters of lesions. Survival times were calculated from the start of treatment.

#### Quality of life

Patient's quality of life was assessed during the trial using the Functional Assessment of Chronic Illness Therapy (FACIT G) (Cella *et al*, 1993) core questionnaire. Overall wellbeing was measured using the Trial Outcome Index (TOI) which is the combined scores of the functional and physical domains with the site specific subscales. Fatigue was measured using the symptom specific subscale for fatigue. Questionnaires were given to patients within 1 week of commencing on the clinical study and at days 7, 14, 21, 42 and 56 following the treatment. Non-parametric analyses were carried out using the Statistical Package for the Social Sciences (SPSS) version 8. The Wilcoxon Signed ranks test was used to measure difference between time points and the Friedman test to measure differences overall.

### Patients

The trial had Local Ethics Committee (LREC), Department of Health Medicines Controls Agency, and Administration of Radioactive Substances Committee (ARSAC) approval. It was performed according to the principles of Good Clinical Practice, under the auspices of Cancer Research UK Phase I/II Clinical Trials Group. Cancer Research UK Drug Development Office monitored the clinical data.

All patients gave written informed consent for the study. The eligibility criteria were unresectable, locally recurrent or metastatic colorectal carcinoma or other CEA expressing tumour; no anti-tumour treatment in the previous 4 weeks; bidimensionally measurable disease by plain X-ray, CT or ultrasound scan; age ⩾18 years; life expectancy ⩾4 months; WHO performance status 0, 1 or 2; and normal haematological, biochemical, renal and hepatic function unless abnormal due to tumour. Pre-treatment serum CEA levels were required to be between 10 μg l^−1^ and 1000 μg l^−1^: if the serum CEA was not raised, then CEA had to be demonstrated by immunohistochemistry on tumour specimens (Boxer *et al*, 1994). Patients were excluded if they had pre-existing HAMA to A5B7, or HACPG2A; the presence of active brain metastasis; if they were a poor medical risk; HIV, Hep B or C positive; or pregnant or lactating.

All patients had an intradermal skin test to the A5CP conjugate performed and would have been excluded if they formed a positive reaction to it. All patients had received previous conventional chemotherapy or radiotherapy, and had either relapsed or showed no response. Preclinical studies indicated the need for the ZD2767P prodrug to be injected into a large bore vein, so all patients had a double lumen Hickman catheter inserted; in most cases this was into the subclavian vein. All patients who received ^131^I-radiolabelled conjugate also received thyroid blocking with potassium iodide 50 mg q.d.s. for 10 days commencing 1 day prior to the administration of the radiolabelled product. Patients could receive metoclopramide as an antiemetic if required after the administration of prodrug.

### Treatment schedule

3000 U m^−2^ of A5CP in 500 ml of 0.9% sodium chloride was given over 2 h on day 0. 250 U of A5CP was radiolabelled with ^131^I 370 MBq and administered as a slow i.v. bolus over 2 min at the end of the infusion. Daily serum CPG2 measurements were performed until the serum CPG2 enzyme levels fell below a pre-determined value. This level was initially 0.20 U ml^−1^ (patients 1, 3–9), however because of early toxicity it was lowered to 0.10 U ml^−1^ (patients 2, 10–22) then 0.05 U ml^−1^ for all subsequent patients. Once this level was achieved, ZD2767P prodrug was administered as three bolus injections, 1 h apart, each over 5 min into a fast running 5% dextrose drip. Patients were then followed up for a period of at least 8 weeks from the time of conjugate administration.

## RESULTS

### Study population

Twenty-seven patients (21 males and six females) were treated with ADEPT. One further patient was recruited but not treated, due to becoming ineligible after registration. The median age was 56 years (range 32–74 years). Twenty-five patients had advanced colorectal cancer, one patient had a CEA expressing carcinoma of the pancreas, and one patient had CEA expressing non-small cell lung cancer. The median number of prior chemotherapy regimens before entering the study was 2 (range 0–7). Fifteen patients (55%) had a WHO performance status of 0, 11 (41%) had a performance status of 1 and one patient (4%) had a performance status of 2 at the time of entry into the study.

### MTD/DLT/toxicity

The dose of A5CP remained fixed throughout the study, with the dose of ZD2767P being escalated until maximum tolerated dose (MTD) was established. The start dose of ZD2767P was 4.9 mg m^−2^×three doses. At 18.63 mg m^−2^ ×three doses, drug-related dose limiting toxicity (DLT) occurred which stopped further dose escalation. This consisted of Grade 4 thrombocytopenia in two out of five patients. One patient at a dose of 18.63 mg m^−2^ also had a prolonged period of neutropenia. The ZD2767P dose was subsequently reduced to 15.5 mg m^−2^ ×three doses in order to establish the MTD.

From dose level 4.9 to 15.5 mg m^−2^ treatment was well tolerated with mainly Grade I–II haematological drug-related adverse events (AEs) (Table 1

**Table 1:**
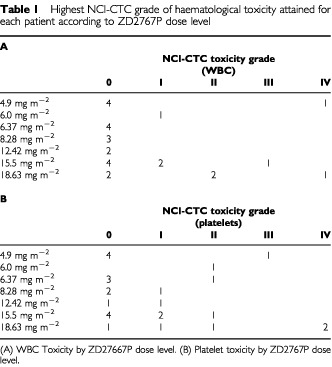
Highest NCI-CTC grade of haematological toxicity attained for each patient according to ZD2767P dose level

). Overall 12 patients experienced Grade I–IV drug related thrombocytopenia, with a median nadir of 4 weeks (27.5 days), and eight patients experienced Grade I–IV drug related leucopenia, with a median nadir of 6 weeks (41.5 days). Other side-effects were mild. Sixteen (59%) patients had grade I drug related nausea and one (4%) patient had grade II nausea. Eleven (41%) patients had grade I or II malaise, which was considered at least possibly drug related. Two (7%) patients had grade III malaise, again considered possibly related to the ADEPT treatment. Six (22%) patients developed a fever in the absence of infection which may have been related to the ADEPT treatment.

Patients who had grade II or greater drug related toxicity with ADEPT were invited to participate in receiving ZD2767P alone, in order to assess its toxicity. Three patients agreed to have ZD2767P alone, and these patients were followed for a further 6–8 weeks. One patient developed Grade 1 nausea after ZD2767P administration. The second patient had no drug-related toxicities. The third patient had a transient Grade 1 leucopenia and neutropenia, which were considered possibly related to ZD2767P.

### A5CP pharmacokinetics

A5CP clearance from the blood was measured by serial CPG2 serum enzyme levels and by clearance of I^131^-radiolabelled A5CP. Three batches of A5CP were used in the trial (Campaign 1, 2 and 4). The β half-life of A5CP by clearance of CPG2 enzyme levels from serum was Campaign 1=8.95 h (spec assay), Campaign 2=11.8 h (cobas) and Campaign 4=15.49 h (cobas). The β half-life of A5CP by clearance of ^131^I-radiolabelled A5CP was Campaign 1=5.77 h, Campaign 2=5.08 h and Campaign 4=11.77 h.

### Serum CPG2 enzyme levels on prodrug day

The median serum CPG2 enzyme level on prodrug day was 0.033 U ml^−1^ (range 0.013–0.152 U ml^−1^) by Spec/Cobas and 0.037 U ml^−1^ (range 0.011–0.180 U ml^−1^) by HPLC. The number of days until prodrug administration was a median of 3 days (range 2–9 days).

### CPG2 enzyme levels in tumour and normal tissue

The median CPG2 enzyme level in tumour biopsies was 0.010 U g^−1^ (range 0–0.208 U g^−1^) on prodrug day (*n*=7) (Table 2a

**Table 2:**
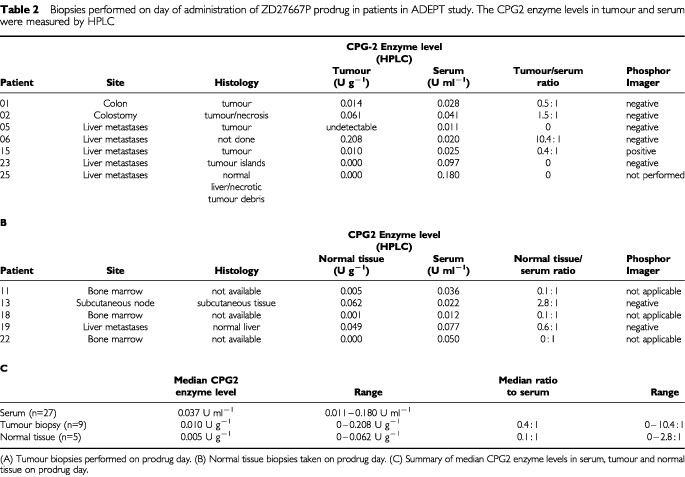
Biopsies performed on day of administration of ZD27667P prodrug in patients in ADEPT study. The CPG2 enzyme levels in tumour and serum were measured by HPLC

). The median CPG2 enzyme level in normal tissue was 0.005 U g^−1^ (range 0–0.062 U g^−1^) (*n*=5) (Table 2b). The median ratio of CPG2 enzyme in tumour : blood on prodrug day was 0.4 : 1 (range 0–10.4 : 1) (Table 2c).

### Gamma camera imaging

**Figure 1:**
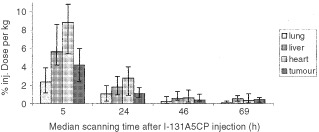
Distribution of ^131^I-radiolabelled A5CP in tumour and normal tissue over time by quantitative SPECT imaging. Each time-point is the median of 18 patients. The error bars include 70% of the data points.

Eighteen patients had SPECT scans performed which were suitable for quantitative analysis. The overall median per cent injected dose per kg of radiolabelled conjugate in the tumour on prodrug day was 0.185 per cent per kg, range 0.0006–1.05 per cent per kg (Figure 1

). The median number of estimated enzyme units in the tumour at the time of prodrug administration was 0.010 U g^−1^, range 0.000037–0.054 U g^−1^. The median uptake of radiolabelled conjugate in heart (estimate for bloodpool) remained higher than in tumour for the 5, 24 and 46 h time-points, and for normal liver it remained higher than tumour at all measured time-points. These results are consistent with the low median tumour to blood ratio of enzyme activity of 0.4 : 1 attained by HPLC assay.

### Immunogenicity

Twenty-six out of 27 patients who received A5CP had sufficient blood samples taken for analysis for immunogenicity. All of these patients developed a positive HAMA response following the single administration of A5CP. The median number of days until developing a positive HAMA was 14 days (range 7–36 days). All patients except one (patient #13) developed a positive HACPG2A response. The overall median number of days until developing a positive HACPG2A was 15 days (range 7–50 days).

### ZD2767P (prodrug) pharmacokinetics

All 27 patients had blood samples taken for pharmacokinetic analysis, however in some patients a full pharmacokinetic profile could not be obtained due to technical problems, in particular haemolysis of blood samples resulting in inaccurate and incomplete data. Table 3

**Table 3:**
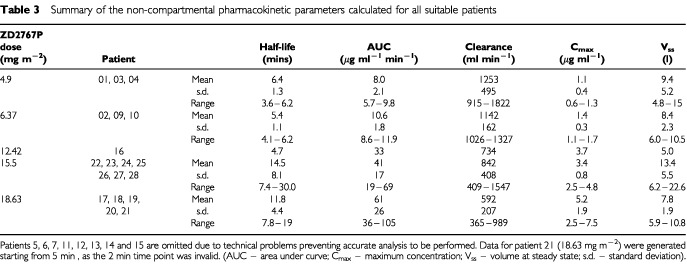
Summary of the non-compartmental pharmacokinetic parameters calculated for all suitable patients

is a summary of the non-compartmental pharmacokinetic values calculated at each dose level for all patients in which reliable analysis could be performed. Average AUC values increased with increasing doses. The mean measured half-life of ZD2767P in plasma at the MTD was 14.5 min. The values for volume of distribution did not appear dose dependent.

Figure 2

**Figure 2:**
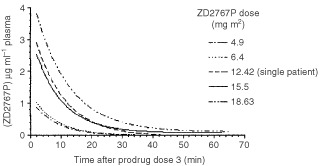
Mean plasma prodrug elimination profile following the third administration of ZD2767P for each dose group. Patients with incomplete pharmacokinetic analyses are excluded. The profile for the 12.42 mg m^−2^ group is that of a single patient.

shows the mean plasma prodrug elimination profiles following the third administration of prodrug, for each dose group of patients. This figure confirms that the prodrug is essentially cleared for all dose levels within 60 min of administration. Figure 3

**Figure 3:**
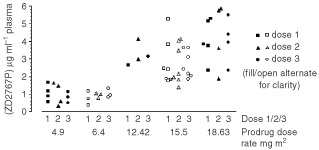
Plasma levels of ZD2767P prodrug measured 2 min after doses 1, 2 and 3. All valid values are included. Individual patients are not identified. Only 4 values are shown for the dose group 18.63 mg m^−2^ after dose three as the pharmacokinetic calculations for one patient were made starting from the 5 min time point.

shows the variation of ZD2767P levels after each of the three administrations of prodrug, at each different prodrug dose. There is no obvious accumulation of prodrug in the 1 h interval between doses.

Three patients who experienced toxicity with full ADEPT were given prodrug alone, and had pharmacokinetic studies performed. Patients 3 (4.9 mg m^−2^) and 19 (18.63 mg m^−2^) had PK parameters that were broadly similar to those when each was given ADEPT (although data for patient 3 were limited).

### Comet assay

The tumour biopsy of one patient (patient 15) had an 80% reduction in tail moment in the comet assay thereby indicating the presence of DNA interstrand cross-links (Webley *et al*, 2001). The circulating lymphocytes in the same patient did not have DNA interstrand cross-links. Eight other patients had comet assays performed on tumour biopsy specimens (*n*=4) and normal tissue (*n*=4). There was no significant reduction in tail moment seen in these samples, or in the circulating blood lymphocytes measured as controls in these eight patients.

### Tumour response

Of the 27 patients, 26 were evaluated for response. There were no objective responses in this study. Three patients had stable disease at day 56.

### Quality of life

Participation in ADEPT did not adversely affect patients' quality of life as measured by the Functional Assessment of Chronic Illness Therapy Trial Outcome Index. There were no significant differences in the amount of fatigue experienced but it could be seen that the fatigue burden varied between patients. Patient's emotional wellbeing was not adversely affected and individual patient scores remained stable.

## DISCUSSION

The purpose of this ADEPT trial was to investigate a new prodrug (bis-iodo phenol mustard, ZD2767P) whose activated form is highly potent and has a short half-life, and to study tumour targeting of A5CP without a clearance system.

The trial was designed in a mechanistic fashion to allow the determination of whether conditions for effective therapy with ADEPT were met. This included the measurement of CPG2 enzyme levels in serum, tumour and normal tissues, ^131^I-labelled conjugate biodistribution studies, immunogenicity assessment, prodrug pharmacokinetic analysis and comet assay for DNA interstrand cross-links.

### Toxicity, efficacy and quality of life

The toxicity of ADEPT consisted mainly of myelosuppression, which proved dose limiting. Myelosuppression occurred with a nadir for thrombocytopenia 4 weeks and a nadir for neutropenia at 6 weeks. One patient developed grade 3 thrombocytopenia and grade 4 neutropenia at the first dose level (4.9 mg m^−2^–Patient no. 3), however reducing the serum enzyme level at which prodrug was given from 0.2 to 0.05 U ml^−1^ meant further dose escalation was possible. DLT was established at a ZD2767P dose of 18.63 mg m^−2^ ×3 administrations and MTD at 15.5 mg m^−2^ ×3 administrations. Other toxicities from ADEPT were mild.

Three patients with drug related toxicity from full ADEPT had ZD2767P prodrug alone administered. This was well tolerated with only a few transient Grade I ‘possibly’ drug related toxicities experienced.

There were no tumour responses but the mechanistic studies (below) indicate the reasons for this and indicate the level of improvement required before efficacy would be expected.

The quality of life data confirmed good tolerability of the treatment as measured by the Functional Assessment of Chronic Illness Therapy Trial Outcome Index. There were no significant differences in the amount of fatigue experienced, but it could be seen that the fatigue burden varied between patients. Patients' emotional wellbeing was also not adversely compromised.

### Mechanistic study

Studying the individual components in terms of their distribution and function facilitates developments of such a complex therapy system. It was also possible to monitor the different components of ADEPT through the course of therapy.

In this ADEPT system the median tumour : blood ratio of antibody-enzyme conjugate at the time of prodrug administration was 0.4 : 1. The amount of enzyme reaching the tumour was a median of 0.010 U g^−1^ (enzyme levels in tumour biopsies, and gamma camera data), too low for optimal prodrug activation in mouse tumour model systems. It has been possible to achieve adequate tumour levels of enzyme (0.5 U g^−1^) with high tumour blood ratios (>10 000 : 1) in a previous clinical study in which a galactosylated clearing antibody was used to accelerate clearance of conjugate from the blood (Napier *et al*, 2000). This previous study did, however, use higher amounts of antibody-enzyme conjugate (10 000   U m^−2^) and the prodrug was given at an earlier timepoint (48 h), so the difference in enzyme levels is at least partially attributable to these factors.

Overall, in this trial, the tumour biopsy CPG2 enzyme data by HPLC and gamma camera dosimetry correlated well, indicating the robust nature of these techniques. A5CP serum half-life was measured by CPG2 enzyme activity and by clearance of radiolabelled A5CP. The CPG2 assays (spectrophotometric/Cobas) are functional measures of antibody-enzyme conjugate, which depend on the presence of intact CPG2. They are clinically relevant as they determine whether prodrug can be safely administered to avoid activation in the peripheral circulation. Measurement of radioactivity clearance may reflect not only intact antibody-enzyme, but also, fragments of antibody-enzyme with radioactivity attached. Radiolabelled material with low or absent enzyme activity may have been potentially metabolised *in vivo*, or damaged by the radiolabelling process, artificially shortening the circulating half-life. The shorter half-life of A5CP obtained by measuring clearance of radioactivity as opposed to functional CPG2 enzyme in this trial is therefore likely to be due to clearance of iodinated breakdown products of ^131^I-radiolabelled A5CP.

Initially a spectrophotometric assay was used to prospectively measure serum CPG2 enzyme levels, and later a Cobas assay was adopted. This change was initiated as a result of sensitivity problems with the spectrophotometric assay at enzyme levels below 0.05 U ml^−1^. The two assays gave similar results above this level. The HPLC assay provides the most accurate measurement of CPG2 enzyme level, however it was not practical to run this assay on a daily basis in a prospective manner to determine CPG2 enzyme levels for treatment in the clinical trial. The HPLC assay was run retrospectively in all patients on the pre-prodrug enzyme level in order to confirm the estimation attained on the spectrophotometric or Cobas assays. A rapid HPLC methotrexate reduction assay has now been developed which will supersede both the spectrophotometric and Cobas assays for future clinical trials.

The formation of HAMA and HACPG2A occurred in all patients except one (Patient no. 13–no HACPG2A formation). CPG2 is of bacterial origin, and is used in ADEPT systems because it has no human equivalent that may lead to endogenous enzyme activation. The A5B7 F(ab)_2_ antibody is murine. The immunogenicity of A5CP limits potential for repeated therapy although immunosuppressants have been used to permit up to three therapies in the same patient in the past (Ledermann *et al*, 1991; Bagshawe *et al*, 1995; Bagshawe and Sharma, 1996; Napier *et al*, 2000).

The pharmacokinetic profile of ZD2767P prodrug was measured in blood by HPLC. The elimination half-life of ZD2767P at MTD was 14.5 min. The active drug has such a short biological half-life that it could not be measured in this study. An indirect measurement instead used a comet assay on cells retrieved from tumour biopsies in order to ascertain the presence of DNA interstrand crosslinks. The active drug is an alkylating agent, and produces cell kill by DNA interstrand crosslink formation, so the presence of crosslinks in the tumour biopsy specimen would indicate that an alkylating agent had been active at the tumour site. Within 30 min of the tumour biopsy specimen being taken, peripheral blood was also taken to look for DNA crosslinks in circulating lymphocytes. Patient no. 15 had a biopsy of a liver metastasis of colorectal cancer. An 80% reduction in comet tail, indicating the presence of DNA interstrand crosslinks was demonstrated. The circulating peripheral lymphocytes were unaffected, showing no evidence of DNA crosslink formation. In this patient, the tumour biopsy specimen also showed evidence of ^131^I conjugate localisation on phosphor imager (Table 2a). This suggests that in this one patient effective antibody localisation and selective prodrug activation may have occurred. This patient had radiological stable disease at 56 days.

In summary, serum, tumour and normal tissue enzyme levels could be monitored by direct measurement on tissue samples and by serial quantitative gamma camera imaging of radiolabelled antibody-enzyme conjugate. Tumour to serum enzyme ratios, where determined, were less than one, which is inadequate for selective prodrug activation in tumour. ZD2767P levels could be measured serially in plasma. Evidence of prodrug activation was obtained in tumour by comet assay and in the blood, indirectly, by the finding of myelosuppression when prodrug was given after antibody-enzyme and by lack of myelosuppression when prodrug was given alone. The universal production of antibody to A5CP prevented repeated therapy.

This study showed that it was practical to formulate and use this relatively unstable prodrug (ZD2767P) as part of ADEPT in the hospital clinical environment and that A5CP conjugate could be given safely and enzyme levels could be monitored and prodrug administered when serum enzyme levels had fallen to a safe level. These are the essential elements of future developments of ADEPT.

This trial has also demonstrated the potential of ZD2767P for use in further ADEPT studies, in which better tumour localisation of antibody-enzyme can be achieved. It has shown also that it is desirable to use a clearance system with the A5CP chemical conjugate, or a better antibody-enzyme targeting system should be developed. These studies provide the basis for rational design of improvements in ADEPT. In particular an antibody-enzyme system is required with more efficient tumour localisation and less immunogenicity.

A new phase I ADEPT study has recently commenced using a recombinant fusion protein of anti-CEA sFv fused to CPG2, in combination with ZD2767P. In preclinical studies the new fusion protein appears to have many of the desirable properties required for use in ADEPT for the future (Sharma *et al*, 2000).
